# Collaborative Optimization of High-Resolution Representation and Miss-Sensitive Supervision for Aero-Engine Micro-Crack Detection

**DOI:** 10.3390/jimaging12070294

**Published:** 2026-07-01

**Authors:** Zixuan Li, Jiaxin Liu, Hongwei Wang, Zhaoming Liu, Feng Zhang, Ning Bai, Jing Hou, Yongliang Yang, Long Cui

**Affiliations:** 1 State Key Laboratory of Robotics and Intelligent Systems, Shenyang Institute of Automation Chinese Academy of Sciences, Shenyang 110016, China; lizixuan@sia.cn (Z.L.); liuzhaoming@sia.cn (Z.L.); zhangfeng@sia.cn (F.Z.); baining@sia.cn (N.B.); ylyang@sia.cn (Y.Y.); cuilong@sia.cn (L.C.); 2 University of Chinese Academy of Sciences, Beijing 100049, China; 3 School of Electrical and Control Engineering, Shenyang Jianzhu University, Shenyang 110168, China; 15953321633@163.com (J.L.); dqhoujing@sjzu.edu.cn (J.H.)

**Keywords:** aero-engine blades, visual inspection, defect detection, computer vision

## Abstract

Aero-engine blades operate under extreme conditions involving high temperature, pressure, rotational speed, and cyclic loads, making them susceptible to surface defects such as micro-cracks. Due to their small scale, weak edges, low contrast, and elongated morphology, micro-cracks are easily affected by metallic reflections, uneven illumination, and complex background textures in borescope images, resulting in high missed-detection rates for conventional detection methods. To address these challenges, this study proposes an improved YOLO11-based framework for aero-engine blade micro-crack detection. The proposed method introduces P1/P2 shallow high-resolution detection branches to enhance the perception of fine crack edges and textures, incorporates Focal Loss to alleviate foreground–background imbalance, applies object-level Tversky Loss to strengthen false-negative constraints, and adopts a hard mining strategy to improve learning for difficult crack samples. Experiments conducted on a real aero-engine borescope image dataset demonstrate that the proposed model achieves a Precision of 0.9981, Recall of 0.9606, F1-score of 0.9790, mAP50 of 0.9781, and mAP50-95 of 0.6938 on an independent test set. Compared with the YOLO11 baseline, the proposed method significantly improves crack detection accuracy, localization quality, and robustness in complex borescope inspection scenarios.

## 1. Introduction

Aero-engine blades operate for long periods under extreme conditions characterized by high temperature, high pressure, high rotational speed, and complex cyclic loads, making them one of the most failure-prone components in aero-engines. Surface defects such as cracks, corrosion, ablation, notches, and coating spallation can significantly degrade structural strength and aerodynamic performance, and may even lead to blade fracture and engine failure, thereby posing serious threats to flight safety [1,2,3]. Among these defects, micro-cracks usually emerge at the early stage of damage evolution and exhibit characteristics such as small scale, elongated morphology, low contrast, and concealed propagation, making them one of the most challenging defect types in aero-engine blade inspection [4,5]. Therefore, developing intelligent micro-crack detection methods with high accuracy and low missed-detection rates is of great significance for improving engine maintenance capability and operational safety.

Traditional blade inspection mainly relies on manual visual inspection and non-destructive testing techniques, including industrial borescopes, eddy current testing, ultrasonic testing, and radiographic inspection. Industrial borescopes can capture internal blade images without disassembling the engine; however, the inspection process still heavily depends on operator experience, resulting in low efficiency, strong subjectivity, and a high risk of missing tiny defects [1,6]. In addition, borescope images are often affected by metallic reflections, local overexposure, uneven illumination, and complex background textures, which further blur micro-crack boundaries and increase the difficulty of automatic detection [7,8].

In recent years, deep learning methods have achieved remarkable success in industrial defect detection. Two-stage detectors such as Faster R-CNN and Mask R-CNN provide high localization accuracy but suffer from relatively slow inference speed, limiting their applicability in real-time inspection scenarios [9,10]. Owing to their end-to-end architecture, fast inference, and deployment convenience, YOLO-series algorithms have been widely applied in industrial surface defect detection and aero-engine blade inspection tasks [11,12,13,14]. Furthermore, attention-based methods, such as DETR and Swin Transformer, enhance global contextual modeling and demonstrate promising performance in complex defect recognition [15,16]. Existing studies on aero-engine blade inspection have explored borescope image enhancement, graph neural network modeling, frequency-domain enhancement, and Transformer-based feature learning [2,7,17,18]. Nevertheless, most existing approaches are designed for general surface defects and remain insufficiently optimized for micro-cracks, which are characterized by small scale, elongated geometry, and weak texture features.

The main challenges in micro-crack detection can be summarized into three aspects. First, micro-cracks occupy only a very small proportion of image pixels, and their shallow edge and texture features are easily weakened during repeated down sampling in YOLO networks, making it difficult for detection heads to produce effective responses. Although conventional P3/P4/P5 feature layers provide strong semantic representations, their limited spatial resolution restricts their ability to preserve crack endpoints, boundaries, and continuity structures [11,19]. Second, micro-cracks belong to typical curvilinear objects with high aspect ratios and strong local continuity. Conventional convolutional detection networks are generally more suitable for blob-like objects and lack sufficient capability to model the topological structures of crack-like targets, often resulting in discontinuous detection and missing edges [5]. Third, the number of normal background regions in blade images is significantly larger than crack regions, leading to severe class imbalance. Conventional cross-entropy or IoU-based losses are easily dominated by background samples, resulting in insufficient recall. In aviation safety scenarios, the cost of missed detection is substantially higher than that of false alarms. Therefore, detection models should place greater emphasis on suppressing false negatives. In this context, asymmetric loss formulations such as Focal Loss and Tversky Loss have been shown to be effective for handling class imbalance and enhancing sensitivity to difficult positive samples [20,21].

To address the challenges of small scale, weak edges, elongated morphology, and high missed-detection cost in aero-engine borescope images, this paper proposes an improved YOLO11-based method for micro-crack detection. Unlike general-purpose object detection approaches, the proposed method is not a simple combination of existing modules; instead, it constructs a task-driven detection framework centered on high-resolution crack perception, miss-sensitive supervision, and hard-crack self-enhanced learning, specifically tailored to the imaging characteristics and safety requirements of micro-crack detection. The main contributions of this paper are summarized as follows:

1. To alleviate the degradation of edge and texture information of micro-cracks in deep networks, a P1/P2 shallow high-resolution crack perception branch is introduced, enabling crack endpoints, edges, and local textures to participate in detection prediction at higher spatial resolution, thereby improving the model’s sensitivity to small-scale cracks.
2. To address the severe imbalance between defect regions and background regions in borescope images, Focal Loss is incorporated as a hard-sample reweighting mechanism to reduce the dominance of easy background samples during training and enhance the model’s attention to low-confidence cracks and cracks under complex backgrounds.
3. Considering that missed detections are more critical than false alarms in aero-engine crack inspection, Tversky Loss is extended to the object-level detection matching process. By assigning higher weights to false negatives, a miss-sensitive constraint mechanism is constructed to improve the recall capability for real crack regions.
4. To improve learning on low-contrast, highly reflective, and easily confused crack samples, a Hard Mining-based self-enhanced learning strategy is designed. Based solely on training-set prediction results, missed cracks, low-confidence cracks, and category-confused cracks are selectively sampled and repeatedly trained to strengthen the model’s learning capability for difficult crack samples.

Compared with existing YOLO-based small-object detection methods, which mainly focus on feature pyramid enhancement, attention mechanisms, or multi-scale fusion, the proposed method is specifically designed for aero-engine micro-crack detection. It constructs a task-driven collaborative framework that jointly considers high-resolution shallow feature representation, miss-sensitive object-level supervision, and hard sample mining based on failure-mode analysis.

## 2. Dataset and Defect Characteristic Analysis

The dataset is a private industrial dataset collected from aero-engine borescope inspection processes. The dataset used in this study was collected from aero-engine borescope inspection images. Borescope inspection is an important non-destructive testing (NDT) technique widely used in aero-engine maintenance. By inserting a borescope through inspection ports into the engine interior, visual inspection of compressor blades and flow-channel surfaces can be performed without engine disassembly, enabling defect screening and condition assessment during in-service maintenance [1].

However, due to the complex internal structure of aero-engines, borescope images are often affected by metallic reflections, local overexposure, shadow occlusion, and motion blur. In addition, complex machining textures and material reflectance characteristics on blade surfaces further reduce the grayscale contrast between micro-cracks and the background, resulting in blurred defect boundaries and discontinuous local structures, which significantly increase the difficulty of automatic detection [2]. Moreover, variations in imaging angles, blade curvature, and surface textures across different regions further challenge the stable extraction of defect features.

The dataset used in this study is a private industrial dataset collected from real aero-engine borescope inspection procedures. It contains 311 original borescope defect images with a resolution of 960 × 720 pixels and 386 valid annotated defect instances, including 185 crack instances and 201 gouge instances. The original images were first divided into training, validation, and test sets at an approximate ratio of 70%, 15%, and 15%, corresponding to 217, 46, and 48 images, respectively. Specifically, the training set contains 128 crack instances and 143 gouge instances, the validation set contains 29 crack instances and 28 gouge instances, and the test set contains 28 crack instances and 30 gouge instances. After the data split, data augmentation was applied only to the training set, including image scaling, horizontal/vertical flipping, random brightness and contrast adjustments, blur processing, and Gaussian noise injection. As a result, the number of training samples used for model learning was increased to 1077, while the validation and test sets were kept unchanged to ensure unbiased model selection and final evaluation. Two representative blade defect categories are included: crack and gouge. Crack defects mainly appear as elongated fatigue cracks or surface cracks characterized by narrow widths, weak edges, and strong continuity. Gouge defects mainly correspond to local scratches, material grooves, or mechanical abrasion on blade surfaces. Both defect types are common high-risk surface defects in aero-engine service environments [3].

YOLO-format annotations were adopted, including defect categories and corresponding bounding-box coordinates. The dataset contains a total of 386 valid defect instances, including 185 crack instances and 201 gouge instances, as summarized in Table 1. Although the two defect categories are relatively balanced at the instance level, crack regions occupy only a very small proportion of image pixels, resulting in severe foreground–background imbalance at the pixel level. This imbalance can cause background regions to dominate gradient updates during training, thereby weakening the network’s ability to learn micro-crack features [20].

Compared with common industrial defects, micro-cracks in aero-engine blades exhibit several unique characteristics. First, cracks belong to typical curvilinear objects with elongated structures and strong directional continuity. Shi [5] pointed out that curvilinear objects differ significantly from conventional blob-like objects in topological representation, making it difficult to preserve their edge and directional information using only deep semantic features. Consequently, conventional object detection networks are prone to edge discontinuity and local missed detection during crack detection. Second, the grayscale contrast between cracks and the background is often weak. In borescope images, metallic reflections, machining textures, and complex illumination conditions further reduce crack boundary visibility, causing blurred edges, partial discontinuities, and low-contrast appearances. In addition, crack targets are generally very small. After multiple convolution and down sampling operations in YOLO-based networks, shallow texture and edge features are easily overwhelmed by high-level semantic features, making it difficult for detection heads to effectively perceive micro-cracks. This is also one of the primary reasons for the relatively low recall of existing general-purpose object detectors in aero-engine crack inspection tasks [13,14].

**Figure 1:**
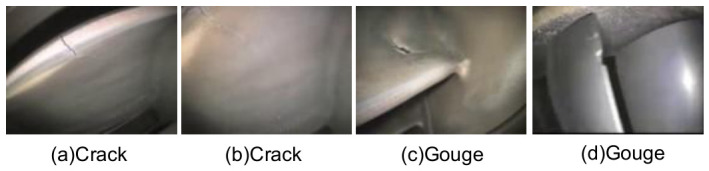
Representative defect detection samples in aero-engine blade borescope images: (**a**,**b**) crack defects with slender and weak-edge characteristics; (**c**,**d**) gouge defects with relatively localized surface damage morphology.

Figure 1 presents several representative crack samples. As shown in the figure, most cracks are distributed along blade edges or curved surface regions. Some cracks exhibit grayscale values highly similar to the surrounding background while also suffering from strong reflection and blur interference. Certain cracks are represented only by extremely weak grayscale variations, and their local structures may even be partially occluded by background textures. These characteristics indicate that aero-engine micro-crack detection is not only a small-object detection problem, but also a weak-texture curvilinear structure detection problem.

Based on dataset analysis, the main challenges of defect detection can be summarized as follows. First, micro-cracks are extremely small, and their edge information is easily degraded during repeated down sampling in deep networks. Second, cracks exhibit elongated curvilinear structures, making it difficult for conventional detection networks to preserve their continuous topological characteristics. Third, borescope images contain significant interference from metallic reflections, shadows, and background textures, which can easily lead to false detections and missed detections. Fourth, some cracks exhibit very low contrast and grayscale values close to the background, resulting in insufficient feature responses. Fifth, severe imbalance exists between crack regions and background regions, causing model training to be dominated by background samples. Finally, aero-engines are typical safety-critical systems, where the cost of missed crack detection is substantially higher than that of false alarms. Therefore, detection models must place greater emphasis on recall performance and false-negative suppression capability [20,21].

Consequently, conventional YOLO detection frameworks cannot directly satisfy the requirements of aero-engine micro-crack detection. Specialized optimization is required in terms of high-resolution feature representation, class imbalance handling, and miss-sensitive detection mechanisms. In the following sections, targeted improvements to the YOLO11 framework are introduced to enhance the detection capability and engineering applicability of the model in complex borescope inspection scenarios.

It should be noted that the dataset used in this study only includes crack and gouge defects, and does not cover other aero-engine defect types. In addition, bounding-box annotations cannot fully represent the curvilinear structure of micro-cracks, which may limit fine-grained geometric interpretation.

## 3. Crack Detection Method

YOLO-based small-object detection methods generally improve detection performance by introducing shallow detection heads, modifying feature pyramid structures, or incorporating attention mechanisms. However, aero-engine blade micro-cracks are fundamentally different from conventional small objects, as they exhibit elongated curvilinear structures, weak texture boundaries, low contrast, and high missed-detection risk. Therefore, simply introducing high-resolution detection layers at the network level may enhance crack edge responses while simultaneously amplifying interference from metallic reflections, machining textures, and pseudo-edges, making stable performance improvement difficult.

The core innovation of the proposed method lies in constructing a collaborative optimization framework that jointly considers network structure, loss supervision, and sample learning for the micro-crack detection task. First, the P1/P2 high-resolution branches establish shallow crack-aware feature pathways for crack endpoints, weak edges, and fine-grained textures, alleviating the information degradation caused by deep feature down sampling. Second, Tversky Loss introduces object-level true positives (TP), false positives (FP), and false negatives (FN) into the detection training process based on IoU matching between predicted and ground-truth boxes, thereby strengthening constraints on missed detections. Third, unlike Focal Loss, which performs online sample reweighting, the proposed Hard Mining strategy explicitly identifies missed cracks, low-confidence cracks, and category-confused cracks through periodic model inference and constructs a hard-sample pool for repeated training.

Compared with conventional OHEM-based methods, the proposed Hard Mining strategy does not perform generic ranking based solely on sample loss values. Instead, it is specifically designed around typical failure modes in aero-engine crack detection. Missed cracks correspond to insufficient recall, low-confidence cracks correspond to weak-response samples, and crack-as-gouge predictions correspond to category confusion. This task-oriented design provides explicit crack-detection semantics and is more suitable for safety-critical micro-crack inspection scenarios.

**Figure 2:**
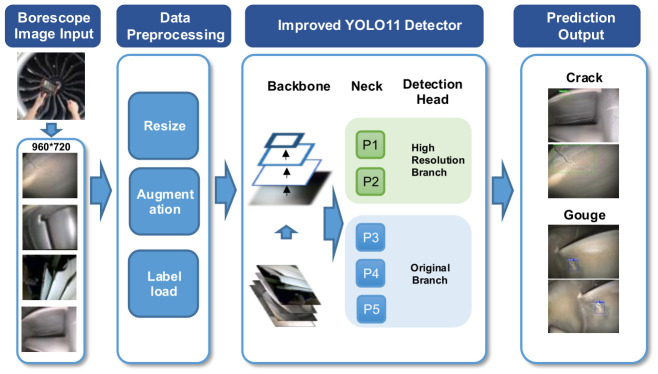
Improved YOLO11 detection framework. The proposed framework enhances micro-crack detection by integrating P1/P2 high-resolution feature branches, Focal-Tversky loss, and hard mining-based difficult sample learning.

The overall framework of the proposed method is illustrated in Figure 2. First, a high-resolution crack perception mechanism is introduced to enhance the representation capability for crack edges, textures, and local structures. Second, a miss-sensitive joint supervision mechanism is constructed to simultaneously alleviate class imbalance and strengthen constraints on false-negative samples. Finally, a hard-crack self-enhanced learning mechanism is incorporated to perform targeted learning on missed and category-confused cracks, thereby improving the robustness and generalization ability of the model in complex borescope inspection scenarios.

### 3.1. High-Resolution Crack Perception

#### 3.1.1. Micro-Crack Feature Analysis

YOLO11 architecture mainly consists of three components: Backbone, Neck, and Detection Head. The Backbone extracts hierarchical image features, the Neck performs multi-scale feature fusion through feature pyramids and path aggregation, and the Detection Head outputs object categories, bounding-box locations, and confidence scores. For conventional object detection tasks, YOLO11 achieves a good balance between detection accuracy and inference speed.

**Figure 3:**
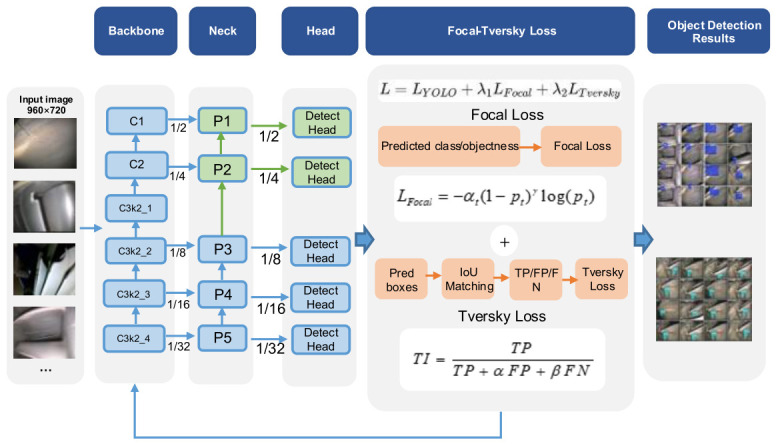
Improved YOLO11 network structure with P1/P2 high-resolution detection branches for enhanced micro-crack feature extraction and multi-scale detection.

However, aero-engine micro-cracks differ significantly from ordinary targets. Micro-cracks occupy only a very small number of pixels and exhibit elongated structures with weak grayscale variations along their boundaries. As illustrated in Figure 3, after multiple convolution and down sampling operations in YOLO-based networks, crack endpoints, texture details, and edge structures contained in shallow features gradually degrade. Conventional YOLO detection branches mainly rely on deep features such as P3, P4, and P5 for prediction. Although these deep features possess strong semantic representation capability, their relatively low spatial resolution limits their ability to preserve local micro-crack structures. Consequently, directly applying the original YOLO11 model to aero-engine micro-crack detection often results in insufficient crack responses, inaccurate boundary localization, and missed detection of tiny cracks.

Let the input borescope image be denoted as *I*, with spatial dimensions *H*×*W* and three channels

(1) $$I\in {\mathbb{R}}^{H\times W\times 3},$$

After hierarchical feature extraction through the backbone network, a multi-scale feature set is obtained

(2) $$F=\{F_{1},F_{2},\ldots ,F_{n}\},$$

where $F_{i}$ represents the feature map generated at the *i*-th stage. As the network depth increases, the spatial resolution decreases while semantic representation capability becomes stronger. For conventional objects, deep semantic information benefits category discrimination; however, for micro-cracks, shallow features containing edge contours, local textures, and grayscale discontinuities are more critical. Therefore, YOLO11 must be adaptively optimized to improve its perception capability for weak-texture small targets.

#### 3.1.2. Shallow High-Resolution Crack Representation

To improve the representation capability for local micro-crack structures, this study introduces P1/P2 high-resolution detection branches into the original YOLO11 framework. By fusing shallow high-resolution texture features with deep semantic features, the network enhances its perception of crack edges, endpoints, and local texture details.

In YOLO-based networks, the feature map at the *i*-th layer can generally be expressed as

(3) $$F_{i}\in {\mathbb{R}}^{H_{i}\times W_{i}\times C_{i}},$$

where *C*_*i*_ denotes the number of feature channels. As network depth increases, feature-map resolution decreases while receptive field size and semantic abstraction capability increase. When crack targets are extremely small, their responses on deep low-resolution feature maps may correspond to only a few pixels or even become overwhelmed by background textures, making stable prediction difficult.

To effectively fuse shallow detail information with deep semantic information, progressive up sampling and feature concatenation are employed to construct high-resolution fused features. For the P2 detection branch, the fusion process is formulated as

(4) $$P2=Concat\left(Up\left(F_{3}\right),\;F_{2}\right),$$

Similarly, the P1 branch is defined as

(5) $$P1=Concat\left(Up\left(P2\right),\;F_{1}\right),$$

where *Up*( ) denotes the up sampling operation and *Concat*(⋅) represents channel-wise feature concatenation. Through this design, deep semantic information is progressively propagated to high-resolution spatial locations, while shallow texture and edge information directly participate in detection prediction, forming crack detection features with both semantic discrimination capability and local-detail perception capability.

The shallow features introduced by P1/P2 are not independently used for prediction; instead, they are fused with deep semantic features through FPN-style aggregation, which helps suppress noise from metallic reflections and machining textures.

Accordingly, the detection feature set is extended to

(6) $$P=\left\{P1,\;P2,\;P3,\;P4,\;P5\right\},$$

Compared with conventional detection structures, the P1/P2 high-resolution branches enable crack edges, endpoints, and local textures to directly enter the detection head at higher spatial resolution, thereby reducing detail information loss caused by repeated down sampling and improving the response capability for tiny and elongated cracks.

#### 3.1.3. Multi-Scale Crack Prediction and Decoupled Detection Head

The proposed method retains the decoupled detection head design of YOLO11, separating classification and bounding-box regression tasks to reduce optimization conflicts between different objectives. In aero-engine blade defect detection, crack and gouge defects differ significantly in morphology, spatial scale, and texture distribution. Excessive parameter sharing between classification and localization branches may lead to optimization interference, thereby reducing classification stability and localization accuracy.

Let the *k*-th candidate bounding box be defined as

(7) $$b_{k}=\left(x_{k},\;y_{k},\;w_{k},\;h_{k}\right),$$

where $x_{k},\;y_{k}$ denotes the center coordinates, $w_{k}$ and $h_{k}$ denote the width and height of the predicted box, respectively. Across all prediction scales, the model output can be represented as

(8) $$Y=\{(B_{k},p_{k},c_{k})|k=1,2,\ldots ,N\}$$

where $B_{k}$, $p_{k}$ and $c_{k}$ denote the bounding box, confidence score, and category prediction of the *k*-th candidate target, respectively, and *N* denotes the total number of candidate targets across all scales.

By introducing the P1/P2 high-resolution detection branches, the model can generate candidate boxes at finer spatial scales, thereby increasing the coverage of candidate regions for micro-cracks and enhancing the detection capability for weak-edge and low-contrast cracks.

Although oriented bounding boxes (OBB) may provide more precise geometric fitting for elongated cracks, this study adopts axis-aligned bounding boxes due to annotation simplicity and consistent comparison with YOLO-based baselines.

### 3.2. Miss-Sensitive Supervision

#### 3.2.1. Hard-Sample Reweighting Based on Focal Loss

In aero-engine borescope images, most regions correspond to normal background areas, while crack and gouge defects occupy only a small proportion of the image. Although the numbers of crack and gouge instances are relatively balanced, background samples still vastly outnumber defect samples from both pixel-distribution and candidate-box perspectives. When conventional cross-entropy loss is directly used for training, optimization tends to be dominated by numerous easy background samples, reducing the model’s attention to weak-feature targets such as micro-cracks.

To alleviate the class imbalance problem, Focal Loss is introduced as a class-balancing optimization term. For a ground-truth class probability *p*_*t*_, Focal Loss is defined as

(9) $$FL(p_{t})=-\alpha {\left(1-p_{t}\right)}^{\gamma }\log(p_{t}),$$

where *α* is the class-balancing factor and *γ* is the modulation factor. When a sample is easily classified, *p*_*t*_ becomes large and its loss contribution is reduced. Conversely, difficult samples receive larger loss weights. Therefore, Focal Loss suppresses the dominance of easy background samples and enables the model to focus more on low-confidence cracks and hard samples under complex backgrounds.

#### 3.2.2. Recall Enhancement Constraint Based on Tversky Loss

Tversky Loss was originally proposed for medical image segmentation, where true positives, false positives, and false negatives are usually computed at the pixel level. However, in object detection tasks, TP, FP, and FN are commonly determined by discrete IoU-threshold assignment between predicted boxes and ground-truth boxes. Such hard binary counting is non-differentiable and therefore incompatible with gradient-based optimization. Since the present study adopts YOLO-format bounding-box annotations rather than pixel-level masks, this paper extends the Tversky concept to object-level detection supervision by constructing differentiable soft approximations of TP, FP, and FN based on prediction confidence and continuous IoU responses.

Let the ground-truth box set and prediction box set be denoted as

(10) $$G=\left\{g_{i}\right\},$$

(11) $$B=\left\{b_{j}\right\},$$

where $g_{i}$ denotes the i-th ground-truth box, $b_{j}\;$ denotes the j-th predicted box, and $p_{i}$ denotes the confidence score of $b_{j}$. For each predicted box and ground-truth box pair, the soft matching response is defined as

(12) $$s_{ij}=p_{i}*IoU(b_{i},g_{i})$$

where $IoU(g_{i},b_{j})$ represents the overlap between the predicted box and the ground-truth box. A prediction contributes more strongly to the soft true-positive term only when it has both high confidence and high spatial overlap with a ground-truth object.

For clarity, the notation used in Equations (10)–(15) is further specified. The index i refers to ground-truth bounding boxes, while index j refers to predicted bounding boxes. The predicted confidence score $p_{j}$ is associated with the j-th predicted box $b_{j}$. All related formulations in Equations (10)–(15) follow this consistent indexing convention to avoid ambiguity in the definition of object-level matching and loss computation.

Based on the soft matching response, the differentiable approximations of true positives, false positives, and false negatives are formulated as

(13) $$TP_{soft}=\sum_{i}\underset{j}{\max}(s_{ij})$$

(14) $$FP_{soft}=\sum_{i}\left(1-maxIoU\left(b_{i},g_{i}\right)\right)p_{i}$$

(15) $$FN_{soft}=\sum_{j}\left(1-\underset{i}{\max}(s_{ij})\right)$$

Here, $TP_{soft}$ measures the confidence-weighted overlap between predicted boxes and ground-truth boxes, $FP_{soft}$ penalizes high-confidence predictions that have weak overlap with any ground-truth box, and $FN_{soft}$ penalizes ground-truth objects that are not sufficiently covered by high-confidence predictions. Unlike discrete TP/FP/FN statistics, these soft terms are constructed before threshold-based post-processing and can serve as differentiable surrogate approximations for gradient-based training.

The object-level Tversky coefficient is then reformulated as

(16) $$TI=\frac{TP_{soft}}{TP_{soft}+\alpha FP_{soft}+\beta FN_{soft}+\varepsilon }$$

where α and β control the penalty weights for false- positive and false-negative responses, respectively, and ε is a small constant used to avoid numerical instability. The corresponding Tversky Loss is defined as

(17) $$L_{Tversky}=1-TI,$$

In this study, the false-negative penalty is assigned a larger weight than the false-positive penalty

(18) β>α

This setting reflects the recall-first optimization principle in aero-engine micro-crack detection, where missed cracks are more critical than false alarms. By increasing the penalty on $FN_{soft}$, the proposed loss encourages the detector to produce stronger responses for real crack regions, thereby reducing the risk of missed detection.

Since the proposed terms are constructed from continuous confidence scores and IoU responses rather than discrete TP/FP/FN counting, the resulting Tversky-style objective acts as a differentiable surrogate loss and remains compatible with end-to-end backpropagation in YOLO training. Compared with conventional IoU-based losses, this asymmetric supervision term provides a more explicit constraint against missed micro-cracks and is therefore suitable for safety-critical aero-engine borescope inspection scenarios.

#### 3.2.3. Focal–Tversky Joint Optimization

Although introducing high-resolution detection branches enhances crack-detail representation, it may simultaneously increase responses to background textures. Focal Loss improves hard-sample learning but provides limited constraints on missed detections, whereas Tversky Loss strengthens false-negative suppression but may become unstable without class-imbalance constraints. Therefore, a Focal–Tversky joint supervision mechanism is further constructed to simultaneously address class imbalance, hard-sample learning, and crack missed-detection suppression during training.

The overall loss function is defined as

(19) $$L_{total}=L_{cls}+\lambda_{1}L_{box}+\lambda_{2}L_{Focal}+\lambda_{3}L_{Tversky},$$

The bounding-box regression loss is expressed as

(20) $$L_{box}=L_{IoU},$$

Rather than simply summing multiple loss terms, the proposed joint loss forms a collaborative optimization mechanism specifically designed for micro-crack detection. The high-resolution crack perception mechanism enhances crack candidate responses, Focal Loss improves the classification weight of difficult cracks under complex backgrounds, and Tversky Loss reduces missed-detection risk. Together, these components enable the model to achieve balanced localization accuracy, classification stability, and crack recall performance in complex borescope inspection scenarios.

### 3.3. Hard-Crack Self-Enhanced Learning

**Figure 4:**
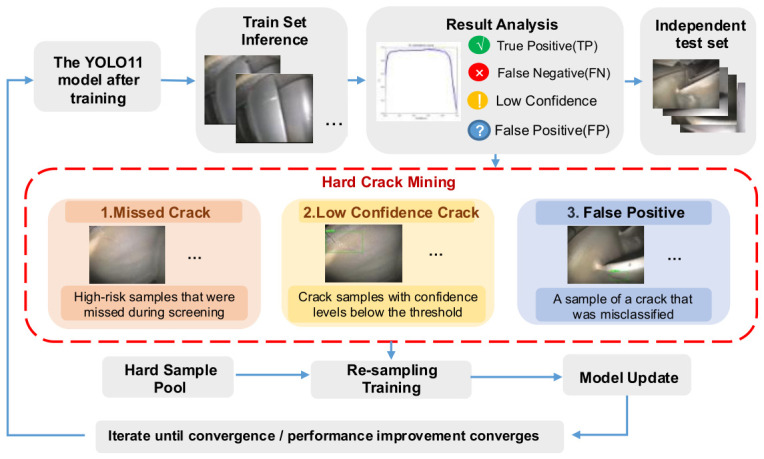
Hard-crack self-enhanced learning framework for reinforcing missed, low-confidence, and misclassified crack samples during training.

Hard crack samples in aero-engine borescope images usually exhibit low contrast, blurred edges, partial occlusion, and strong reflection interference. Under random sampling, the model tends to preferentially learn salient cracks with clear edges and weak background interference, while paying insufficient attention to low-confidence and weak-response cracks. To further improve robustness in complex borescope scenarios, a hard-crack self-enhanced learning mechanism is proposed to explicitly learn typical failure modes during training. The overall structure is illustrated in Figure 4.

According to model predictions, hard crack samples are categorized into three failure modes:

(1) Missed cracks;
(2) Low-confidence cracks;
(3) Crack-as-gouge confusion samples.

These three categories correspond to deficiencies in recall capability, confidence estimation, and semantic discrimination ability, respectively.

Let the ground-truth crack box be g_*i*_ and the predicted box set be *B*. The maximum matching IoU is defined as

(21) $$IoU_{max}(g_{i})=\max_{b_{j}\in B}IoU(g_{i},b_{j}),$$

If the maximum matching IoU is lower than a predefined threshold, the crack is regarded as undetected

(22) $$IoU_{max}(g_{i})<\tau_{iou},$$

For matched prediction boxes with confidence lower than a threshold, the crack is defined as a low-confidence hard sample

(23) $$p_{i}<\tau_{c},$$

If a crack target is predicted as the gouge category, it is considered a category-confused hard sample

(24) $$c_{i}=gouge,$$

The final hard-sample set is represented as

(25) $$H=H_{miss}\cup H_{low}\cup H_{confuse},$$

During training, repeated sampling or sample duplication is adopted to increase the occurrence frequency of hard crack samples. Let the weight of normal samples be 1 and the repetition coefficient of hard samples be *r*. The sample weight is defined as

(26) $$w_{i}=\left\{\begin{array}{ll} r, & x_{i}\in H\\ 1, & otherwise \end{array}\right.,$$

The corresponding sampling probability is

(27) $$P(x_{i})=\frac{w_{i}}{\sum_{j}w_{j}},$$

The overall algorithmic procedure is summarized in Table 2.

Compared with Online Hard Example Mining (OHEM), which selects samples dynamically based on instantaneous loss within mini-batches, the proposed Hard Mining strategy operates at the data level by explicitly constructing a hard sample pool based on inference-driven failure modes, including missed cracks, low-confidence detections, and category-confused samples. This design enables iterative re-sampling across training cycles, making it fundamentally different from loss-level reweighting methods such as OHEM and static oversampling strategies.

The hard sample pool is dynamically updated in each mining iteration to avoid overfitting to fixed background patterns.

The proposed strategy differs fundamentally from Focal Loss. Focal Loss performs online reweighting at the loss-function level, whereas Hard Mining explicitly constructs a hard-sample pool at the data level and repeatedly learns from exposed failure modes. These two mechanisms enhance the model’s hard-crack learning capability from the perspectives of loss supervision and sample distribution, respectively.

Due to data confidentiality constraints and the industrial nature of the dataset, detailed numerical statistics of hard samples in each category are not reported. Instead, the three failure modes (missed cracks, low-confidence cracks, and crack-as-gouge cases) are explicitly defined and used to construct the hard sample pool, ensuring full methodological reproducibility.

## 4. Results and Discussion

To verify the effectiveness of the proposed collaborative optimization framework for aero-engine blade micro-crack detection, experiments were conducted on a real aero-engine borescope dataset. Multiple ablation studies were performed to systematically analyze the contribution of each module, including the P1/P2 high-resolution detection branches, Focal Loss, Tversky Loss, and the Hard Mining strategy. The experiments focus on micro-crack detectability, bounding-box localization accuracy, robustness under complex backgrounds, and crack recall performance.

### 4.1. Experimental Settings

Experiments were conducted on a Windows 11 platform equipped with an NVIDIA RTX 4000 Ada GPU, a 13th Gen Intel(R) Core(TM) i9-13900K CPU, and 32 GB RAM. The proposed models were optimized using the AdamW optimizer.

All input images were resized to 640 × 640. The initial learning rate was set to 0.01, and the weight decay was set to 0.0005. The batch size was fixed at 8, and all models were trained for 200 epochs. A fixed random seed of 42 was used to ensure reproducibility.

During inference, the confidence threshold was set to 0.70, and the IoU threshold for evaluation was set to 0.70.

For the proposed Hard Mining strategy, the following parameters were adopted: HARD_EXTRACT_CONF = 0.02, HARD_EXTRACT_IOU_MATCH = 0.30, and HARD_EXTRACT_LOW_CONF = 0.50.

To ensure fair comparison, all experiments adopted the same data split, training epochs, and an independent test set. Since this study focuses on weak-target perception and missed-detection suppression in aero-engine micro-crack detection, particular attention was paid to detection stability in complex backgrounds, low-contrast crack regions, and elongated crack regions. All models were evaluated under the same inference threshold to avoid additional interference caused by confidence-threshold differences.

### 4.2. Evaluation Metrics

All metrics are computed at the object level based on IoU matching, rather than pixel-wise classification accuracy.

Therefore, the primary quantitative comparisons in this study focus on YOLO-series baselines and variants trained and evaluated under identical experimental settings, while two-stage and Transformer-based detectors are discussed mainly as literature-based references rather than primary experimental baselines due to possible differences in datasets, annotations, and evaluation protocols.

Precision, Recall, F1-score, mAP50, and mAP50-95 were used as the main evaluation metrics. Precision measures the proportion of correctly detected defects among all predicted defects, while Recall measures the proportion of real defects successfully detected by the model. F1-score is the harmonic mean of Precision and Recall, reflecting the overall balance between false-positive control and missed-detection suppression.

mAP50 denotes the mean average precision at an IoU threshold of 0.5, reflecting the overall detection capability. mAP50-95 is calculated over multiple IoU thresholds and is therefore more sensitive to bounding-box localization quality.

### 4.3. Results

To evaluate the effects of different modules, six experimental settings were designed:

(1) YOLO11 detection baseline;
(2) YOLO11 segmentation baseline;
(3) YOLO11 with P1/P2 high-resolution detection branches;
(4) P1/P2 with Focal Loss;
(5) P1/P2 with Focal–Tversky joint supervision;
(6) The complete model with P1/P2, Focal Loss, Tversky Loss, and Hard Mining. The results are shown in Table 3.

The results show that the proposed collaborative framework significantly improves the overall performance of aero-engine micro-crack detection. Compared with the YOLO11 detection baseline, the complete model improves Precision from 0.8909 to 0.9981, Recall from 0.8598 to 0.9606, F1-score from 0.8751 to 0.9790, and mAP50-95 from 0.6121 to 0.6938. These results demonstrate that the proposed method improves not only defect detectability, but also localization accuracy, background suppression, and robustness in complex borescope scenarios.

After introducing only the P1/P2 high-resolution detection branches, Recall increases from 0.8598 to 0.9345, and crack mAP50-95 improves from 0.5310 to 0.6180. This indicates that shallow high-resolution features effectively enhance the model’s perception of micro-cracks. Since cracks are small weak-texture targets, their edge and texture information can be gradually degraded during repeated down sampling in conventional YOLO structures. The P1/P2 branches allow crack edges and local textures to participate in feature representation at higher spatial resolutions, improving response strength and candidate-region coverage.

However, the improvement in Precision is limited when only high-resolution branches are added. This suggests that shallow high-resolution features may also introduce more background texture responses, including metallic reflections, machining traces, shadows, and pseudo-edge structures. Therefore, high-resolution feature enhancement alone is insufficient; stronger supervision is required to distinguish weak cracks from complex background interference.

When Focal Loss is further introduced, Precision increases to 0.9912 and F1-score reaches 0.9689, indicating that class imbalance is effectively alleviated. In borescope images, background regions greatly outnumber crack regions. Standard cross-entropy loss tends to be dominated by easy background samples, whereas Focal Loss reduces their contribution and increases the influence of low-confidence hard samples. As a result, the model becomes more sensitive to weak cracks under complex backgrounds.

For the YOLO11 segmentation baseline, since pixel-level annotations are not available in this dataset, bounding-box annotations are directly converted into rectangular mask regions. The segmentation head is trained using these generated masks to enable a fair comparison with detection-based variants.

To further evaluate the computational efficiency of the proposed method, we report the number of parameters, model size, and inference speed for all ablation variants. As shown in the experiments, the number of parameters increases gradually from 2.58M (baseline model) to 2.91M (full model with P1/P2, Focal Loss, Tversky Loss, and Hard Mining), indicating that the proposed modules introduce only a marginal computational overhead.

Similarly, the model size increases from 5.307 MB to 6.782 MB across different configurations, demonstrating that the enhanced detection performance is achieved with a relatively lightweight increase in storage cost.

In terms of inference efficiency, the full model achieves a real-time processing speed of 70.22 FPS on an NVIDIA RTX 4000 Ada GPU, which satisfies the requirements for real-time aero-engine borescope inspection. These results demonstrate that the proposed method maintains a good balance between detection accuracy and computational efficiency. After adding Tversky Loss, Recall further increases from 0.9477 to 0.9744, and F1-score improves to 0.9743. This demonstrates that Tversky Loss effectively strengthens the constraint on missed crack detections. By assigning a higher penalty to false negatives, the model is encouraged to focus more on potential crack regions rather than solely optimizing bounding-box overlap. This is particularly important for aero-engine inspection, where missed detections are more critical than false alarms.

With the Hard Mining strategy, the model achieves the highest F1-score and mAP50-95. The mAP50-95 reaches 0.6938, representing an additional improvement of approximately 9.83% over the P1/P2 + Focal + Tversky model. This indicates that targeted learning of difficult samples further improves localization quality and detection stability in complex scenarios.

**Figure 5:**
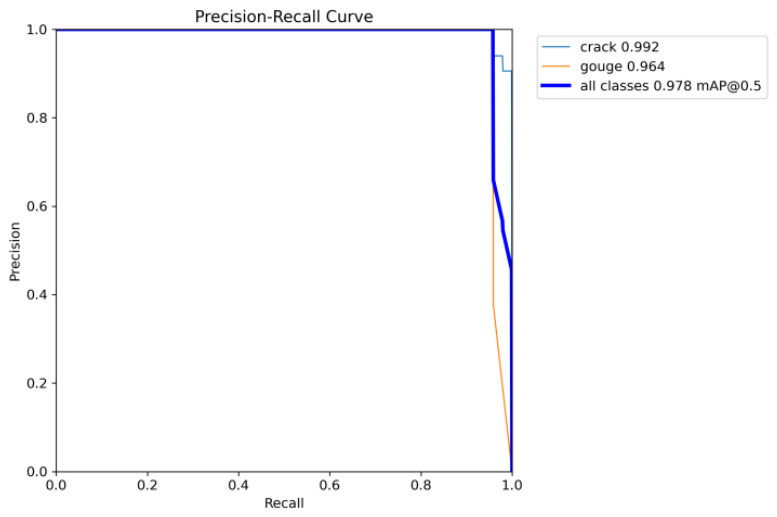
Evaluation curves of the improved YOLO11 model.

The Precision–Recall (PR) curves of the proposed model at an IoU threshold of 0.5 are shown in Figure 5. Under this evaluation setting, the crack category achieves an AP@0.5 of 0.992, while the gouge category achieves an AP@0.5 of 0.964, resulting in an overall mAP@0.5 of 0.9781. It should be noted that AP@0.5 mainly reflects category-level detection capability under a relatively loose localization criterion, whereas mAP@0.5:0.95 provides a stricter and more comprehensive evaluation of bounding-box localization quality across multiple IoU thresholds. As reported in Table 3, the complete model achieves an overall mAP@0.5:0.95 of 0.6938, with the crack-category mAP@0.5:0.95 reaching 0.6869. Therefore, the AP@0.5 value of 0.992 should be interpreted as the crack-category PR-curve result at IoU = 0.5, rather than as the comprehensive mAP@0.5:0.95 metric.

As shown in the figure, both PR curves at IoU = 0.5 remain close to the upper-right corner, indicating that the model maintains high precision and recall under the mAP@0.5 evaluation setting. In particular, the crack category exhibits a more stable PR curve, demonstrating the effectiveness of the proposed method for weak-texture and elongated micro-crack detection. These results further verify the strong detection capability and background suppression performance of the proposed framework.

**Figure 6:**
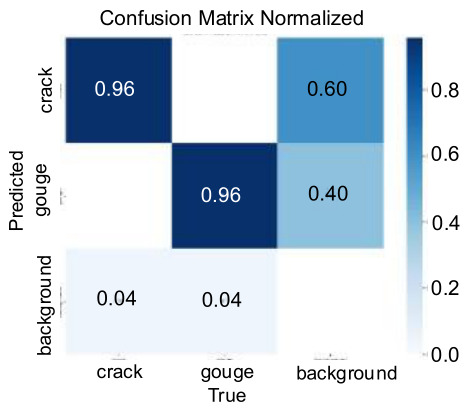
Confusion matrix of the improved YOLO11 algorithm.

The confusion matrix in Figure 6 shows that the model achieves high recognition accuracy for both crack and gouge defects, with most samples correctly classified. This indicates that the model effectively learns the texture and morphological differences between different blade damage types.

The predictions for the crack category are mainly concentrated along the diagonal, demonstrating strong recognition capability for micro-cracks. The gouge category also achieves high classification accuracy, indicating that the model can distinguish elongated crack-like structures from block-like gouge damage. In addition, the confusion between background and defect categories is limited, suggesting effective background suppression under complex borescope conditions. This further confirms the effectiveness of Focal Loss and Hard Mining in reducing interference from complex background textures.

Nevertheless, a small number of off-diagonal errors remain, indicating that some low-contrast or blurred-edge cracks may still be misclassified. This is mainly due to metallic reflections, machining textures, and local shadows in borescope images, which can make some crack regions visually similar to the background.

**Figure 7:**
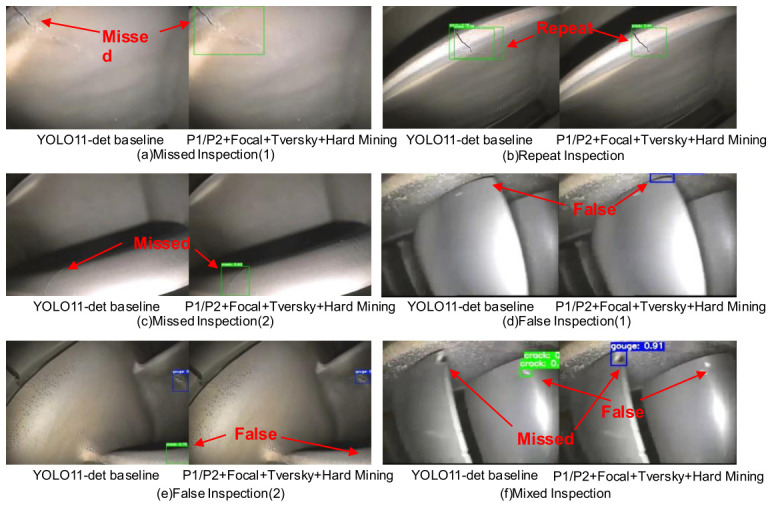
Testing results of the improved YOLO11 model. (**a**) Comparison of missed crack detection in complex weak-texture regions; (**b**) repeated crack inspection results under different confidence responses; (**c**) comparison of missed inspection for low-contrast micro-cracks; (**d**) false inspection comparison in complex background regions; (**e**) false detection suppression results for pseudo-defect structures; (**f**) mixed inspection results containing both missed and false detections in challenging borescope images.

The qualitative detection results are shown in Figure 7.

The proposed Hard Mining strategy continuously emphasizes missed cracks, low-confidence cracks, and category-confused cracks, enabling the model to repeatedly learn weak crack structures in complex backgrounds. It also reinforces the effects of high-resolution feature enhancement and joint supervision. Specifically, the high-resolution branches enhance crack-detail representation, the Focal–Tversky supervision optimizes gradient distribution for difficult and missed cracks, and Hard Mining maintains the model’s attention to complex crack samples during later training. Therefore, the proposed method forms a collaborative optimization loop of feature enhancement, supervision constraint, and hard-sample targeted learning, leading to overall performance improvement in aero-engine micro-crack detection.

**Figure 8:**
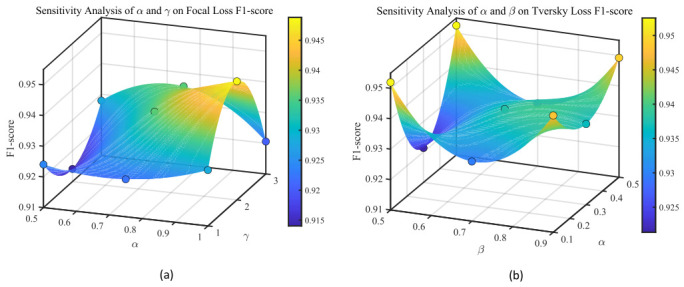
Sensitivity analysis of the Focal–Tversky supervision mechanism. (**a**) Influence of the Focal Loss hyperparameters α and γ on the validation F1-score; (**b**) influence of the Tversky Loss hyperparameters α and β on the validation F1-score. The results illustrate the impact of class-balancing, hard-sample focusing, and false-negative penalty weighting on micro-crack detection performance.

To investigate the influence of hyperparameter selection on the proposed Focal–Tversky supervision mechanism, sensitivity analyses were conducted for the key parameters of Focal Loss and Tversky Loss. The F1-score on the validation set was adopted as the evaluation metric, and the results are illustrated in Figure 8.

Figure 8a presents the sensitivity analysis of the Focal Loss parameters α and γ. It can be observed that the model’s performance is influenced by both parameters. When γ is relatively small, the suppression effect on easy samples is insufficient, limiting the model’s ability to focus on difficult crack instances. As γ increases, the F1-score gradually improves and reaches its maximum when α ≈ 0.9 and γ ≈ 2.5. However, excessively large γ values lead to over-emphasis on hard samples and may reduce optimization stability, resulting in performance degradation. These results indicate that an appropriate balance between class weighting and hard-sample focusing is essential for micro-crack detection.

Figure 8b shows the sensitivity analysis of the Tversky Loss parameters α and β. The results demonstrate that assigning a larger penalty to false negatives generally leads to better performance in the aero-engine crack detection scenario. The highest F1-score is achieved when α≈0.1 and β≈0.9, indicating that stronger false-negative suppression is beneficial for safety-critical inspection tasks. As β increases from lower values, the model exhibits improved crack sensitivity and recall capability. Nevertheless, when β becomes excessively dominant, the performance gain gradually saturates, suggesting that overly aggressive false-negative weighting may introduce additional false-positive responses. Overall, the analysis confirms that the asymmetric penalty design of the proposed Tversky formulation is well aligned with the recall-oriented requirements of micro-crack detection.

Based on the above analysis, α = 0.9 and γ = 2.5 were selected for Focal Loss, while α = 0.1 and β = 0.9 were adopted for Tversky Loss in all subsequent experiments.

## 5. Conclusions

This study investigated the problem of micro-crack detection in aero-engine blade borescope images. Considering the characteristics of micro-cracks, including small scale, weak edge responses, elongated morphology, complex background interference, and high missed-detection cost, an improved YOLO11-based method was proposed for aero-engine blade micro-crack detection. 

Experimental results show that the crack-category mAP50-95 increases from 0.5310 to 0.6180 after introducing the P1/P2 branches, demonstrating that shallow high-resolution features effectively enhance the perception capability for small-scale and weak-texture crack targets. This result further indicates that relying solely on deep semantic features is insufficient to preserve the elongated structures and edge details of micro-cracks in aero-engine borescope images, whereas high-resolution shallow features play a critical role in improving crack detectability.

To address insufficient learning of low-contrast cracks, highly reflective cracks, and easily confused crack samples in real borescope images, a Hard Mining strategy was further designed for targeted learning of missed cracks, low-confidence cracks, and misclassified cracks. The hard-sample pool was constructed solely based on predictions from the training set, without using validation or test samples, thereby avoiding data leakage and ensuring objective evaluation. Experimental results show that the complete model achieves a Precision of 0.9981, Recall of 0.9606, F1-score of 0.9790, mAP50 of 0.9781, and mAP50-95 of 0.6938 on the independent test set. In particular, the crack-category mAP50-95 reaches 0.6869, representing a 29.36% improvement over the YOLO11 detection baseline, while the gouge-category mAP50-95 reaches 0.7007. These results verify the effectiveness of the proposed method for aero-engine micro-crack detection.

Although promising results were achieved, several limitations remain. First, the dataset mainly contains crack and gouge defects and does not yet cover other aero-engine blade defects such as ablation, corrosion, and coating spallation. Therefore, the generalization capability of the model in more diverse multi-defect scenarios still requires further validation. Second, this study adopts bounding-box annotations, whereas micro-cracks usually exhibit elongated curvilinear structures that cannot be fully represented by rectangular boxes. Consequently, the true topology and edge extension characteristics of cracks are not completely captured. In addition, although the Hard Mining strategy improves learning for difficult crack samples, the selection thresholds and repeated-sampling weights still require further optimization to achieve a better Precision–Recall balance.

Future work can be conducted in the following directions:

1. Expanding aero-engine borescope defect datasets to include more defect categories, operating conditions, and background environments;
2. Introducing crack-skeleton constraints, curvilinear structural priors, or detection–segmentation fusion mechanisms to better characterize the geometric morphology of micro-cracks;
3. Further optimizing hard-sample selection thresholds and repeated-sampling weights to achieve improved Precision–Recall balance;
4. Combining lightweight network design and inference acceleration strategies to improve deployment capability on practical borescope inspection devices and aero-engine maintenance systems.
5. Including comprehensive comparisons with YOLOv10, RT-DETR, and Transformer-based detectors to further validate the generalizability of the proposed method.
6. Addressing the limitation of statistical robustness analysis, future work will conduct multiple independent training runs under different random seeds and report the mean ± standard deviation of evaluation metrics. In addition, statistical hypothesis testing (e.g., t-test) will be introduced to rigorously evaluate the significance of performance improvements and further improve the reliability and reproducibility of the proposed method under stochastic training conditions.

## Figures and Tables

**Table 1:** Description of defect instances.

Category	Description	Legend	Amount
Crack	A linear opening that can easily be seen and which can cause the material to break	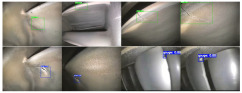	185
Gouge	A large rough cut of large depth with some removal of material caused because a sharp object has hit the part		201

**Table 2:** Hard mining-based hard-crack self-enhanced learning procedure.

**Input:** Training set *D*_*train*_, stage model *M*, IoU threshold *τ*_*iou*_, low-confidence threshold *τ*_*low*_, repetition coefficient *r*	
**Output:** Enhanced training set *D*’_*train*_	
Step	Procedure
1	Initialize model *M*
2	For t = 1 to T, use model *M* to perform inference on the training set *D*_*train*_
3	For each ground-truth crack target, compute the maximum IoU *m*_*i*_ with the prediction set
4	If *m*_*i*_ < *τ*_*iou*_, label the sample as missed crack.
5	If *m*_*i*_ ≥ *τ*_*iou*_ and conf < *τ*_*low*_ ,label the sample as low-confidence crack.
6	If the predicted category is gouge while the ground-truth category is crack, label the sample as crack-as-gouge.
7	Add the identified hard samples into the hard-sample pool *H*.
8	Perform repeated sampling or sample duplication on *H* according to repetition coefficient *r*.
9	Update training set *D*_*train*_ ← *D*’_*train*_ and update model *M*
10	Obtain the enhanced training set *D*’_*train*_ for subsequent model training.

**Table 3:** Ablation results on the independent test set.

No.	Method	Precision	Recall	mAP@0.5	mAP@0.5:0.95	F1	Crack mAP@0.5:0.95	Gouge mAP@0.5:0.95
1	YOLOv11-det baseline	0.8909	0.8598	0.9549	0.6121	0.8751	0.5310	0.6932
2	YOLOv11-seg baseline	0.9350	0.9409	0.9753	0.6165	0.9379	0.4735	0.4501
3	P1/P2	0.8993	0.9345	0.9610	0.6291	0.9166	0.6180	0.6756
4	P1/P2 + Focal	0.9912	0.9477	0.9829	0.6104	0.9689	0.6065	0.6734
5	P1/P2 + Focal + Tversky	0.9743	0.9744	0.9849	0.6317	0.9743	0.6212	0.6824
6	P1/P2 + Focal + Tversky + Hard Mining	0.9981	0.9606	0.9781	0.6938	0.9790	0.6869	0.7007

## Data Availability

The data presented in this study are available on request from the corresponding author.
